# Prognostic Value of Cardiac Magnetic Resonance Imaging in Chronic Aortic Regurgitation: A Systematic Review and Meta-Analysis

**DOI:** 10.31083/j.rcm2412359

**Published:** 2023-12-25

**Authors:** Jin-Rong Ni, Wen-Long Xin, Yuan Hu, Shi-Dong Liu, Jin-Kui Li, Zun-Hui Wan, Jun-Qiang Lei

**Affiliations:** ^1^The First Hospital (First Clinical Medical School) of Lanzhou University, 730000 Lanzhou, Gansu, China; ^2^Department of Cardiovascular Surgery, The First Hospital of Lanzhou University, 730000 Lanzhou, Gansu, China; ^3^Intelligent Imaging Medical Engineering Research Center of Gansu Province, 730000 Lanzhou, Gansu, China; ^4^Accurate Image and Collaborative Innovation International Scientific and Technological Cooperation Base of Gansu Province, 730000 Lanzhou, Gansu, China; ^5^Gansu Province Clinical Research Center for Radiology Imaging, 730000 Lanzhou, Gansu, China; ^6^Department of Radiology, The First Hospital of Lanzhou University, 730000 Lanzhou, Gansu, China

**Keywords:** aortic valve insufficiency, magnetic resonance imaging, prognosis, heart, follow-up studies

## Abstract

**Background:**

Chronic aortic regurgitation (AR) is a common valvular disease characterized by an overload of left ventricular volume and pressure. Accurate assessment of the heart from all angles is crucial for effective clinical management and prognostic evaluation of AR patients. As an advanced imaging technique, cardiac magnetic resonance (CMR) has become the gold standard for assessing cardiac volume and function. Accordingly, this study aimed to evaluate the prognostic value of CMR in chronic AR.

**Methods:**

EMBASE, Cochrane Library, PubMed, and Web of Science were searched for clinical studies published between inception and July 19, 2022. Only studies that used CMR to assess patients with chronic isolated AR and provided prognostic data were included.

**Results:**

For our analysis, 11 studies, which involved 1702 subjects and follow-up periods of 0.6–9.7 years, were eligible. We identified 13 CMR-related parameters associated with AR prognosis. With aortic valve surgery as the outcome, we estimated the pooled hazard ratios (HRs) for four of these parameters: aortic regurgitation fraction (ARF), aortic regurgitation volume (ARV), left ventricle end-diastolic volume (LVEDV), and LV end-systolic volume (LVESV). The pooled HR for ARF was found to be 4.31 (95% confidence interval [CI]: 1.12–16.59, *p* = 0.034), while that for ARV was 3.88 (95% CI: 0.71–21.04, *p* = 0.116). Additionally, the combined HRs of LVEDV and LVESV were estimated to be 2.20 (95% CI: 1.04–4.67, *p* = 0.039) and 3.14 (95% CI: 1.22–8.07, *p* = 0.018), respectively.

**Conclusions:**

The assessment of ARF, LVEDV, and LVESV via CMR has significant prognostic value in predicting the prognosis of AR patients with aortic valve surgery as an endpoint. It is recommended to consider using multi-parameter CMR in the clinical management of AR patients for timely interventions and effective prognostic evaluation.

## 1. Introduction

Chronic aortic regurgitation (AR) is a common valvular disease characterized by an overload of left ventricular (LV) volume and pressure [1, 2]. The prevalence of AR is 5% among people aged <50 years and can reach 16% in the elderly population (aged ≥70 years) [1]. Although AR progresses slowly, it reaches an annual mortality rate of 10%–20% once symptoms appear [3]. Despite the clear benefit of timely surgery, many patients undergo operations late in the disease course, when they show advanced symptoms and high rates of heart failure and ventricular dysfunction [4].

As an important cardiovascular imaging technique, cardiac magnetic resonance (CMR) imaging provides accurate data on LV function, size, and volume, as well as valve morphology, aortic regurgitant volume (ARV), and aortic regurgitant fraction (ARF) in AR patients [5, 6]. The new guidelines for the management of valvular heart disease suggest that CMR should be used when the echocardiographic images are poor, and the measured value or AR grade is inconsistent with the clinical status of the patients [7, 8]. In the past decade, most studies have focused on the consistency between CMR and echocardiography in evaluating AR, and many studies on AR prognosis have used only echocardiography [9, 10, 11]. With the advances in CMR technology, new imaging markers, such as global longitudinal strain (GLS), late gadolinium enhancement (LGE), and extracellular volume (ECV), have been gradually applied to AR [12, 13]. Therefore, the role of CMR in the management of AR patients and the prognostic values of CMR-related parameters should be clarified. Accordingly, this study aimed to assess the prognostic value of CMR in chronic AR through a systematic review and meta-analysis.

## 2. Methods

This study is reported according to the Preferred Reporting Items for Systematic Reviews and Meta-analyses (PRISMA) statement [14] and the published recommendations [15]. The detailed protocol is accessible at PROSPERO (CRD42022311827) [16, 17].

### 2.1 Data Sources and Search Strategy

We systematically searched EMBASE, Cochrane Library, PubMed, and Web of Science for relevant clinical studies published between inception and July 19, 2022. Subject words were combined with free words, and the search strategies were developed and adapted for each database (Detailed search strategies were provided in the **Supplementary Materials**). For unpublished trials, we searched Clinical Trials.gov and the trial registers on the World Health Organization International Clinical Trials Registry Platform. We also reviewed the references of the included studies and other systematic reviews and meta-analyses to obtain a comprehensive list of included studies.

### 2.2 Study Selection

Studies were selected based on the following inclusion criteria: (1) The original study included patients with aortic valve regurgitation, (2) used CMR, and (3) provided prognostic information related to CMR parameters. The following studies were excluded: Studies (1) on animals or <10 patients, (2) on patients who had other forms of valvular heart disease or underwent heart-surgery treatment (including transcatheter aortic valve replacement), (3) that used qualitative data without evaluating quantitative CMR techniques or did not use modern imaging sequences, such as steady state free precession (SSFP) imaging, and (4) duplicate studies (in which case, the latest or the one with the largest sample size was selected). Two reviewers (JRN and YH) independently screened for eligible studies. Disagreements were resolved by consensus. If consensus could not be reached, a third reviewer (SDL) was referred to for arbitration.

### 2.3 Data Extraction

Two reviewers (JRN and YH) independently extracted data as per a predefined data extraction sheet. The following variables were extracted from the included studies: first author, journal and year of publication, study design, study population, sample size, age, male/female ratio, CMR equipment information and technical methods, quantitative parameters, follow-up period, endpoint, clinical events, statistical methods, effect sizes, and adjustment variables. The extracted data were cross-checked, and disagreements were resolved via discussion or referral to a third reviewer (YH).

### 2.4 Quality Assessment

In this review, the Quality in Prognosis Studies (QUIPS) tool was used to assess the methodological quality of the included studies [18, 19]. Two reviewers (JRN and WLX) independently evaluated QUIPS items and critically appraised each of the bias domains. All disagreements were resolved by consensus.

### 2.5 Data Synthesis and Statistical Analysis

Only parameters that used the same outcome endpoint and were found in ≥3 studies were evaluated via meta-analysis. Pooled hazard ratios (HRs), odds ratios (ORs), and risk ratios (RRs), with the corresponding 95% confidence intervals (CIs), were calculated. A random effects model was selected a priori given the heterogeneity in study design across the included studies. Statistical heterogeneity among the studies was explored using the *$I^{2}$* statistic. A Galbraith plot was used to determine the source of high heterogeneity in the CMR parameters. Egger’s test was used to evaluate the publication bias when the CMR parameters were described in ≥3 articles. Sensitivity analysis was conducted by recalculating pooled HRs after excluding each article once. All the statistical analyses were performed using STATA/SE version 15.1 (Stata Corp, College Station, TX, USA). *p* < 0.05 was considered to indicate statistical significance.

## 3. Results

### 3.1 Search Results

**Fig. 1.**
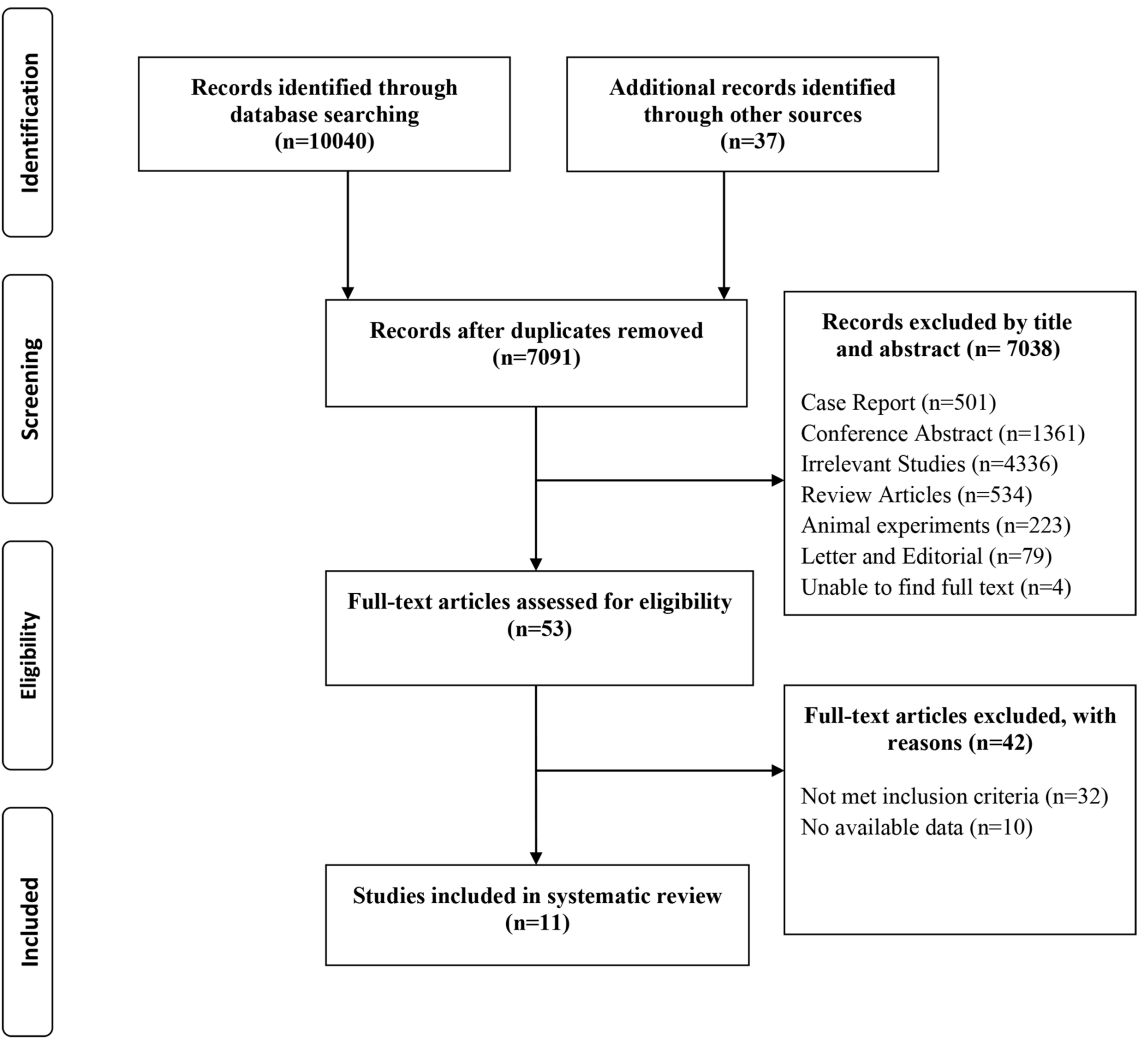
**PRISMA flowchart of study selection**.

The strategy to search for and screen relevant studies is presented in Fig. 1. Electronic and manual searches of the reference lists retrieved 10,077 records. After removing duplicates and primary screening of titles and abstracts, 53 studies were selected for full-text review. Finally, 11 studies [20, 21, 22, 23, 24, 25, 26, 27, 28, 29, 30], which involved 1702 patients, were included in our systematic review.

### 3.2 Study Characteristics

Detailed information about the included 11 studies is presented in Table 1 (Ref. [20, 21, 22, 23, 24, 25, 26, 27, 28, 29, 30]). The articles were all published between 2012 and 2022. There were four studies from the USA, two from Spain, and one each from China, Germany, Belgium, the Czech Republic, and the United Kingdom. Study sample sizes ranged from 29 to 392, and seven studies had sample sizes exceeding 100. The 11 studies included two multi-center prospective studies [28, 30], one dual-center retrospective study [25], two single-center retrospective studies [21, 23], and six single-center prospective studies [20, 22, 24, 26, 27, 29]. In all the included studies except for two [29, 30] that did not report the gender ratio, the majority of patients were male (66%–93%). Seven studies reported the presence of bicuspid aortic valves (BAVs) [21, 22, 25, 26, 28, 29, 30]. The follow-up period of the included studies ranged from 0.6 to 9.7 years. The original studies excluded any lost patients; therefore, no subjects in this study were lost.

**Table 1.** **Baseline characteristics of the included studies**.

First author	Year	Journal	Country	Study design	Population	N	BAV (%)	Age (year)	Male (%)	Equipment	Slice thickness	Scanning sequence
Vejpongsa *et al*. [20]	2022	JACC Cardiovasc Imaging	USA	Prospective (Single center)	AR (LVEF ≥50%)	390	NR	59.2 ± 15.9	265 (67.9)	1.5/3 T Siemens	4–6 mm	SSFP, PC, PSIR
Zheng *et al*. [21]	2021	Eur Radiol	China	Retrospective (Single center)	AR (Stage B–D)	166	46 (27.7)	52 ± 13	147 (88.6)	1.5 T Siemens	NR	SSFP, PC, PSIR
Senapati *et al*. [22]	2021	JACC Cardiovasc Imaging	USA	Prospective (Single center)	AR (NYHA I II III)	177	73 (41.2)	58.0 (47.0–68.0)	117 (66.1)	1.5/3 T Siemens	6 mm	SSFP, PC, PSIR, MOLLI
Fernández-Golfín *et al*. [23]	2021	Eur Radiol	Spain	Retrospective (Single center)	AR (NYHA I IV)	55	NR	60 ± 16.9	40 (80.0)	1.5 T Philips	8 mm	SSFP, PC
Faber *et al*. [24]	2021	Int J Cardiovasc Imaging	Germany	Prospective (Single center)	AR (NYHA I II III)	50	NR	52.4 (38.7–62.5)	35 (70.0)	1.5 T Siemens	5 mm	SSFP, GRE
											8 mm	
Postigo *et al*. [25]	2020	Eur Heart J Cardiovasc Imaging	Spain	Retrospective (Two centers)	AR (Asymptomatic)	197	69 (35.0)	57 (39–71)	160 (81.0)	1.5 T Philips, GE	6 mm	SSFP, PC
											8 mm	
Malahfji *et al*. [26]	2020	JAHA	USA	Prospective (Single center)	AR (Moderate/severe)	392	101 (25.8)	62 (51–71)	306 (78.1)	1.5/3 T Siemens	6 mm	SSFP, PC, PSIR
Seldrum *et al*. [27]	2019	J Cardiothorac Vasc Anesth	Belgium	Prospective (Single center)	AR (NYHA I II)	29	NR	46 ± 12.0	27 (93.0)	1.5 T Philips	NR	SSFP, PC
Kočková *et al*. [28]	2019	J Clin Med.	Czech Republic	Prospective (Three centers)	AR (Moderate-severe/Severe asymptomatic)	104	79 (76.7)	44.4 ± 13.2	89 (85.4)	1.5 T Siemens	6 mm	SSFP, PC, PSIR, MOLLI
											8 mm	
Harris *et al*. [29]	2017	Am J Cardiol	USA	Prospective (Single center)	AR (Asymptomatic)	29	19 (65.5)	47.1 ± 14.6	NR	1.5 T Philips	NR	SSFP, PC
Myerson *et al*. [30]	2012	Circulation	UK	Prospective (Four centers)	AR (Moderate/severe asymptomatic)	113	43 (38.1)	49.0 ± 17.1	NR	1.5 T Philips/Siemens	NR	SSFP, PC

N, number; AR, aortic regurgitation; LVEF, left ventricular ejection fraction; NYHA, New York Heart Association; NR, not report; BAV, bicuspid aortic valve; SSFP, steady-state free-precession; PC, phase-contrast imaging; PSIR, phase-sensitive inversion recovery; MOLLI, modified-look locker inversion recovery; T, Tesla; GRE, gradient echo.

### 3.3 Methodological Evaluation

Methods of image acquisition and post-analysis varied across the studies. All the studies used 1.5 Tesla (T) CMR to scan the heart, and three of them [20, 22, 26] also used 3.0 T CMR. Phase-contrast imaging was used in all the studies. Five studies [20, 21, 22, 23, 26, 28] used phase-sensitive inversion recovery sequences, and the majority used SSFP cine imaging. Among the included studies, one study [23] assessed LV dysfunction and prognosis in AR patients via CMR-feature tracing (CMR-FT)—derived multidirectional strains, whereas another two studies [22, 28] assessed LV remodeling based on ECV fraction by using modified-look locker inversion recovery sequences. For image post-processing, each study used software compatible with the scanning equipment.

**Supplementary Table 1** provides details of the QUIPS quality-assessment items and risk-of-bias assessments. All the included studies had low-to-moderate bias risks. Although all the included studies provided detailed information about the enrolled subjects, CMR protocol, and prognostic follow-up period, some studies did not set comprehensive endpoints, and the confounding factors in these studies were not controlled in the original data analysis.

### 3.4 Prognostic Evaluation

**Fig. 2.**
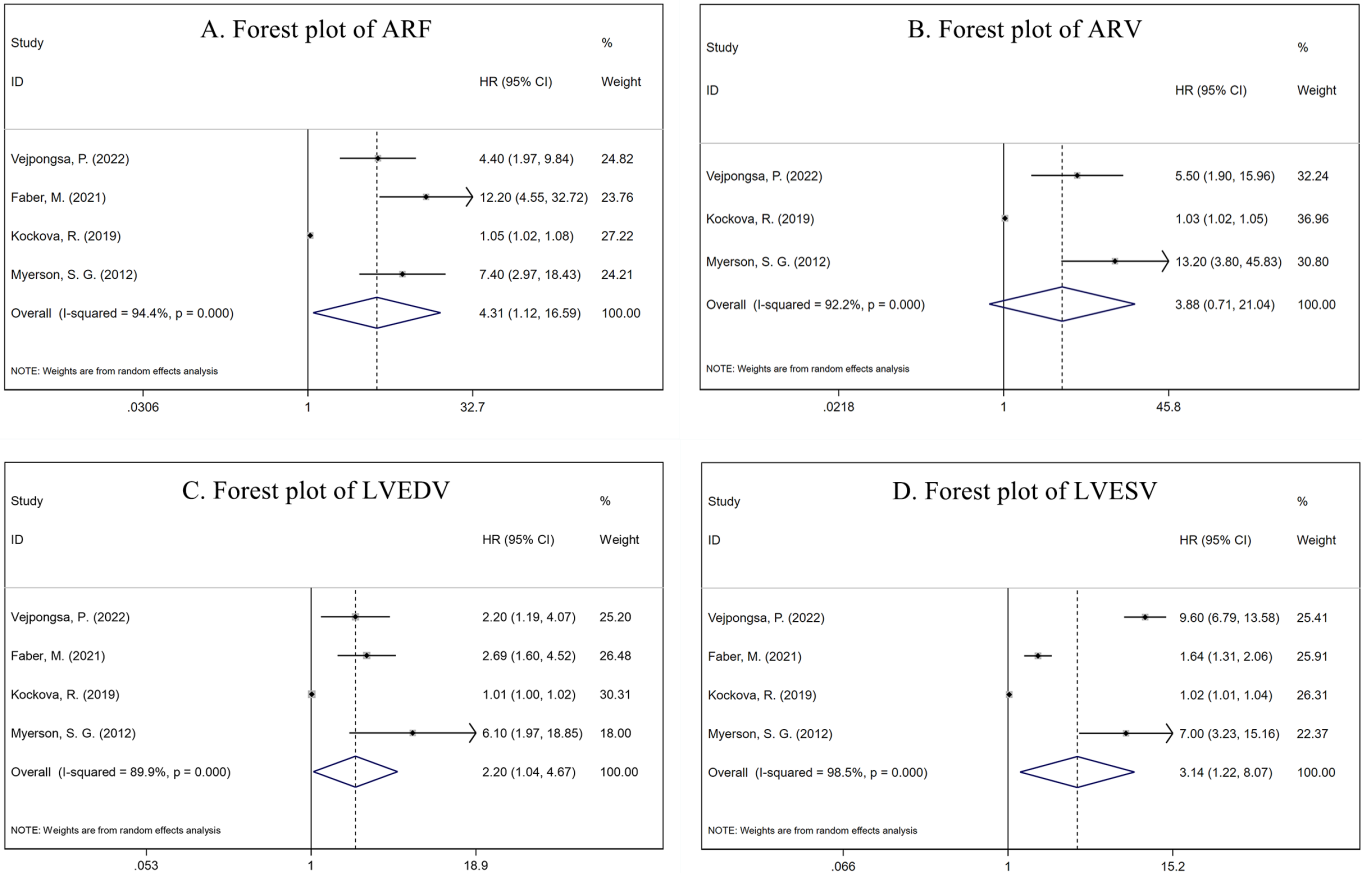
**Forest plot of cardiac magnetic resonance (CMR) parameters for predicting aortic valve surgery**. (A) Pooled HR of ARF was 4.31 (95% CI: 1.12–16.59, *p* = 0.034). (B) Pooled HR of ARV was 3.88 (95% CI: 0.71–21.04, *p* = 0.116). (C) Pooled HR of LVEDV was 2.20 (95% CI: 1.04–4.67, *p* = 0.039). (D) Pooled HR of LVESV was 3.14 (95% CI: 1.22–8.07, *p* = 0.018). ARF, aortic regurgitation fraction; ARV, aortic regurgitation volume; LVEDV, left ventricular end-diastolic volume; LVESV, left ventricular end-systolic volume; HR, hazard ratio.

All-cause mortality [26, 27], intervention via aortic valve surgery [20, 24, 28, 30], and composite endpoints [21, 22, 23, 25] including the above two items and hospitalization for heart failure were the three endpoints of this study. The detailed follow-up information about the included studies is summarized in Table 2 (Ref. [20, 21, 22, 23, 24, 25, 26, 27, 28, 29, 30]). A total of 13 prognosis-related CMR parameters and their details are shown in Table 3 (Ref. [20, 21, 22, 23, 24, 25, 26, 27, 28, 29, 30]) for all the included articles. Finally, we included four studies [20, 24, 28, 30], involving asymptomatic patients with aortic regurgitation (AR) for data synthesis. By utilizing aortic valve surgery as the ultimate endpoint measure, we conducted a meta-analysis to combine data associated with four parameters and derive the final estimated value. Our analysis revealed that the pooled HR of ARF was 4.31 (95% CI: 1.12–16.59, *p* = 0.034). Additionally, we found that the combined HR of ARV was 3.88 (95% CI: 0.71–21.04, *p* = 0.116), and the combined HRs of LV end-diastolic volume (LVEDV) and LV end-systolic volume (LVESV) were determined to be 2.20 (95% CI: 1.04–4.67, *p* = 0.039) and 3.14 (95% CI: 1.22–8.07, *p* = 0.018), respectively. The corresponding forest plots are shown in Fig. 2.

The meta-analysis results also revealed high heterogeneity (*$I^{2}$* > 70%) among the original studies used for our data synthesis. Galbraith plots were generated to investigate the origins of heterogeneity, revealing that three studies [20, 24, 30], excluding Kočková et al.’s [28], were potential sources of heterogeneity (**Supplementary Fig. 1**). Despite the high heterogeneity among the studies, the sensitivity analysis confirmed that the combined HR results of the four parameters (ARF, ARV, LVEDV and LVESV) had good stability (**Supplementary Fig. 2**). Funnel plots obtained using Egger’s test showed a clear publication bias (**Supplementary Fig. 3**). Although several studies have utilized composite endpoints as outcome measures, the specific definitions vary, hampering a meta-analysis on these composite endpoints. Due to the large differences among the selected studies, we could not conduct a subgroup analysis.

## 4. Discussion

This study is the first systematic review focusing on the prognostic values of CMR parameters in isolated chronic AR. By reviewing the existing clinical studies, we summarized the CMR methods and aprameters that can provide effective information for the prognostic evaluation of AR patients and sorted out the existing research directions. Although transthoracic echocardiography is the most commonly used imaging modality in AR, CMR has unique advantages due to its accuracy and repeatability in assessing cardiac volume and function [31, 32, 33]. Recent guidelines [7, 8] consider CMR complementary to echocardiography, but the role of CMR cannot be replaced. Since improving the prognosis in AR is the ultimate goal of optimization of the clinical management, it is essential to assess the prognostic values of CMR parameters.

Recently, studies have shown that CMR is superior to echocardiography for evaluating chronic AR severity [34]. CMR directly measures aortic blood flow, thereby enabling accurate quantification of AR volume and regurgitation fraction and consequently offers unique advantages [34]. It is crucial to fully recognize the importance of accurately quantifying ARV and ARF in clinical practice. The majority of the studies we included focused on ARV and ARF. The meta-pooled results of our study suggest that ARF is a significant parameter in predicting the outcome of aortic valve surgical intervention (HR: 4.31, *p* = 0.034). Although the combined results of ARV showed no statistical significance (HR: 3.88, *p* = 0.116), this observation does not negate the value of ARV in the prognosis of AR. Many scholars have noted that following the guideline-recommended criteria (ARF >50% and ARV >60 mL) may cause delayed intervention in AR. In our included studies, the ARF threshold ranged from 32% to 37%, and the ARV threshold ranged from 38 mL to 50 mL, which were lower than the guideline levels. The earliest one among the included studies [30] proposed that ARF >33% can be considered as an indicator of aortic valve surgery, and the latest multicenter study [20] proposed that the optimal ARF and ARV thresholds are 35% and 38 mL, respectively. These findings not only reaffirm the significance of CMR-derived ARF and ARV for prognosticating AR patients but also indicate the necessity of redefining the corresponding thresholds.

**Table 2.** **Follow-up data of included studies**.

First author	Year	Follow-up (year)	N	Endpoint	Events
Vejpongsa *et al*. [20]	2022	2.1 (0.6–4.5)	390	AVS (replacement/reconstruction)	73 AVS.
Zheng *et al*. [21]	2021	4.7 (3.6–6.2)	166	Composite outcome (all-cause mortality, hospitalization for HF)	45:7 HF, 38 death, 28 Cardiovascular death.
Senapati *et al*. [22]	2021	2.5 (1.07–3.56)	177	Composite outcome (death and AVR)	58:49 AVR,12 death (3 death after AVR).
Fernández-Golfín *et al*. [23]	2021	1.9 (1.5–2.5)	55	Composite outcome (all-cause mortality, AVS, cardiovascular mortality, hospitalization for HF)	16:14 AVS, 1 HF, 1 death.
Faber *et al*. [24]	2021	5.1 (NR)	50	AVS (replacement/reconstruction)	16 AVS.
Postigo *et al*. [25]	2020	2.75 (1.1–5.3)	197	Composite outcome (AVS, hospitalization due to HF, cardiovascular death)	76:6 HF, 70 AVS, 0 death.
Malahfji *et al*. [26]	2020	2.69 (0.81–5.79)	392	All-cause mortality	51 Death.
Seldrum *et al*. [27]	2019	6.83 (2.41–9.67)	29	All-cause mortality	2 Death.
Kočková *et al*. [28]	2019	1.6 (0.81–2.47)	104	AVS (replacement/reconstruction)	20 AVS.
Harris *et al*. [29]	2017	4.4	29	Composite outcome (AVS, hospitalization for HF)	5 AVS.
Myerson *et al*. [30]	2012	2.6 ± 2.1	113	AVS (replacement)	39 AVR.

N, number; AVS, aortic valve surgery; HF, heart failure; AVR, aortic valve replacement; NR, not report.

**Table 3.** **CMR parameters and outcomes of the included studies**.

Parameters	First author	Year	Adjustment degree	HR	95% CI	*p*	Endpoint	Adjusted variables	Notes
**Aortic regurgitation fraction (ARF)**									
[20]	Vejpongsa, *et al*.	2022	Multivariate analysis	4.40	2.0–10.0	<0.001	AVS	Not Report	ARF >35%
[21]	Zheng, *et al*.	2021	Multivariate analysis	1.02	1.0–1.04	0.030	Composite outcome	Not report	
[22]	Senapati, *et al*.	2021	Multivariate analysis	1.71	1.41–2.07	<0.001	Composite outcome	Age, Sex	
[23]	Fernández-Golfín, *et al*.	2021	Univariate analysis	1.02	0.99–1.05	0.167	Composite outcome	NA	
[24]	Faber, *et al*.	2021	Univariate analysis	12.2	4.56–32.8	<0.001	AVS	NA	Standard sequence
[25]	Postigo, *et al*.	2020	Multivariate analysis	1.69	1.41–2.03	<0.001	Composite outcome	Age, sex, and comorbidity	ARF per 10%
[26]	Malahfji, *et al*.	2020	Univariate analysis	1.08	0.95–1.23	0.200	All-cause mortality	NA	ARF per 5%
[27]	Seldrum, *et al*.^#^	2019	Univariate analysis	0.99	0.94–1.06	0.960	All-cause mortality	NA	RR
[28]	Kočková, *et al*.	2019	Multivariate analysis	1.05	1.02–1.08	<0.001	AVS	Volumes or indices	
[29]	Harris, *et al*.	2017	Univariate analysis	1.10	1.02–1.19	0.038	Composite outcome	NA	
[30]	Myerson, *et al*.	2012	Multivariate analysis	7.40	3.00–18.6	<0.001	AVS	Not Report	
**Aortic regurgitant volume (ARV)**									
[20]	Vejpongsa, *et al*.	2022	Multivariate analysis	5.50	1.90–16.0	0.009	AVS	Not Report	ARV >38 mL
[23]	Fernández-Golfín, *et al*.	2021	Univariate analysis	1.01	0.99–1.02	0.160	Composite outcome	NA	
[26]	Malahfji, *et al*.	2020	Univariate analysis	0.96	0.90–1.02	0.260	All-cause mortality	NA	ARV per 5 mL
[27]	Seldrum, *et al*.^#^	2019	Univariate analysis	1.03	0.98–1.07	0.270	All-cause mortality	NA	RR
[28]	Kočková, *et al*.	2019	Multivariate analysis	1.03	1.01–1.04	<0.001	AVS	Volumes or indices	
[29]	Harris, *et al*.	2017	Univariate analysis	1.46	1.10–1.94	0.044	Composite outcome	NA	ARV per 10 mL
[30]	Myerson, *et al*.	2012	Multivariate analysis	13.2	3.80–45.8	<0.001	AVS	Not Report	
**Left ventricular ejection fraction (LVEF)**									
[22]	Senapati, *et al*.	2021	Multivariate analysis	1.02	0.86–1.20	0.860	Composite outcome	Age, Sex	
[23]	Fernández-Golfín, *et al*.	2021	Univariate analysis	0.88	0.79–0.98	0.017	Composite outcome	NA	
[24]	Faber, *et al*.	2021	Univariate analysis	0.49	0.34–0.70	<0.001	AVS	NA	
[25]	Postigo, *et al*.	2020	Multivariate analysis	0.51	0.37–0.69	<0.001	Composite outcome	Age, sex, and comorbidity	LVEF per 10%
[26]	Malahfji, *et al*.	2020	Univariate analysis	0.97	0.95–0.98	<0.001	All-cause mortality	NA	
[27]	Seldrum, *et al*.^#^	2019	Univariate analysis	0.89	0.74–1.06	0.200	All-cause mortality	NA	RR
[29]	Harris, *et al*.	2017	Univariate analysis	0.91	0.73–1.14	0.800	Composite outcome	NA	
**Left ventricular mass (LVM)**									
[20]	Vejpongsa, *et al*.	2022	Multivariate analysis	2.10	1.10–3.60	0.020	AVS	Not Report	LVM >186 g
[30]	Myerson, *et al*.	2012	Univariate analysis	3.20	1.60–6.50	0.020	AVS	NA	
**Left ventricular mass index (LVMi)**									
[23]	Fernández-Golfín, *et al*.	2021	Univariate analysis	1.01	0.98–1.03	0.586	Composite outcome	NA	
[26]	Malahfji, *et al*.	2020	Univariate analysis	1.00	0.99–1.01	0.580	All-cause mortality	NA	
[27]	Seldrum, *et al*.^#^	2019	Univariate analysis	1.06	1.00–1.12	0.040	All-cause mortality	NA	RR
**Left ventricular end-diastolic volume (LVEDV)**									
[20]	Vejpongsa, *et al*.	2022	Multivariate analysis	2.20	1.20–4.10	0.009	AVS	Not Report	LVEDV >220 mL
[24]	Faber, *et al*.	2021	Univariate analysis	2.69	1.60–4.52	<0.001	AVS	NA	
[25]	Postigo, *et al*.	2020	Multivariate analysis	1.15	1.06–1.25	<0.001	Composite outcome	Age, sex, and comorbidity	LVEDV per 25 mL
[28]	Kočková, *et al*.	2019	Multivariate analysis	1.01	1.00–1.01	0.036	AVS	MRI ARF	
[29]	Harris, *et al*.	2017	Univariate analysis	1.37	1.05–1.78	0.110	Composite outcome	NA	LVEDV per 10 mL
[30]	Myerson, *et al*.	2012	Multivariate analysis	6.10	2.00–19.1	0.002	AVS	Not Report	
**Left ventricular end-diastolic volume index (LVEDVi)**									
[23]	Fernández-Golfín, *et al*.	2021	Univariate analysis	0.99	0.95–1.03	0.571	Composite outcome	NA	
[26]	Malahfji, *et al*.	2020	Univariate analysis	1.00	1.00–1.01	0.020	All-cause mortality	NA	
[27]	Seldrum, *et al*.^#^	2019	Univariate analysis	1.08	1.01–1.16	0.110	Composite outcome	NA	RR
[28]	Kočková, *et al*.	2019	Multivariate analysis	1.01	1.00–1.03	0.033	AVS	MRI ARF	
**Left ventricular end-systolic volume (LVESV)**									
[20]	Vejpongsa, *et al*.	2022	Univariate analysis	9.60	5.6–11.20	<0.001	AVS	NA	LVESV >81 mL
[24]	Faber, *et al*.	2021	Univariate analysis	1.64	1.30–2.05	<0.001	AVS	NA	
[28]	Kočková, *et al*.	2019	Univariate analysis	1.02	1.00–1.03	0.017	AVS		
[30]	Myerson, *et al*.	2012	Univariate analysis	7.00	3.20–15.0	<0.001	AVS	NA	
**Left ventricular end-systolic volume index (LVESVi)**									
[26]	Malahfji, *et al*.	2020	Univariate analysis	1.01	1.00–1.02	0.004	All-cause mortality	NA	
[27]	Seldrum, *et al*.^#^	2019	Univariate analysis	1.14	1.02–1.27	0.020	All-cause mortality	NA	RR
[28]	Kočková, *et al*.	2019	Univariate analysis	1.03	1.01–1.06	0.014	AVS		
[29]	Harris, *et al*.	2017	Univariate analysis	3.03	1.02–9.03	0.280	Composite outcome	NA	LVESVi per 10 ${\mathrm{mL/m}}^{2}$
**Left ventricular end-systolic diameter index (LVESDi)**									
[22]	Senapati, *et al*.	2021	Multivariate analysis	1.38	0.61–3.13	0.440	Composite outcome	Age and sex	
[27]	Seldrum, *et al*.^#^	2019	Univariate analysis	1.20	0.92–1.57	0.180	All-cause mortality	NA	RR
**Left ventricular end-diastolic diameter index (LVEDDi)**									
[27]	Seldrum, *et al*.^#^	2019	Univariate analysis	1.36	0.98–1.87	0.060	All-cause mortality	NA	RR
**Late gadolinium enhancement (LGE)**									
[21]	Zheng, *et al*.	2021	Multivariate analysis	1.93	1.03–3.81	0.040	Composite outcome	Not Report	
[26]	Malahfji, *et al*.	2020	Univariate analysis	3.62	2.62–6.36	<0.001	All-cause mortality	NA	
**Extracellular volume index (ECVi)**									
[22]	Senapati, *et al*.	2021	Multivariate analysis	1.34	1.09–1.64	0.010	Composite outcome	Age and sex	
**Global longitudinal strain (GLS)**									
[23]	Fernández-Golfín, *et al*.	2021	Multivariate analysis	1.11	0.91–1.34	0.086	Composite outcome	LVEDVi, LVEF, LAVi, ARF	
**Global radial strain (GRS)**									
[23]	Fernández-Golfín, *et al*.	2021	Multivariate analysis	0.90	0.83–0.98	0.001	Composite outcome	LVEDVi, LVEF, LAVi, ARF	
**Global circumferential strain (GCS)**									
[23]	Fernández-Golfín, *et al*.	2021	Multivariate analysis	1.26	1.04–1.52	<0.001	Composite outcome	LVEDVi, LVEF, LAVi, ARF	

^#^Seldrum, S., *etc*. only reported RR in their research, which is hereby noted. LAVi, left atrium volume index; HR, hazard ratio; RR, relative risk; CI, confidence interval; NA, not applicable; AVS, aortic valve surgery; CMR, cardiac magnetic resonance; MRI, magnetic resonance imaging.

The current guidelines [7, 8] emphasize the importance of monitoring LV size and function in AR patients and suggest critical thresholds for intervention, which are based on echocardiographic measurements of linear dimensions and LVEF in asymptomatic patients. Multiple scholars have stated that guideline-based indications might cause poor prognosis in patients with severe AR and that the threshold should be modified [11, 35, 36]. Despite the higher reproducibility and accuracy of cardiac volume data in tracking changes in patients with AR, the existing guidelines do not include cardiac volume parameters as factors for initiating clinical intervention, whether through echocardiography or CMR. Our study results also demonstrated that LVEDV (HR: 2.20, *p* = 0.039) and LVESV (HR: 3.14, *p* = 0.018) have meaningful prognostic value in guiding surgical intervention for AR patients. Theoretically, indexing cardiac volume parameters by using body surface area may be more accurate than using LVEDV and LVESV. However, due to the limited number of studies currently available, clinical prognostic value of LVEDV index (LVEDVi) and LVESV index (LVESVi) in the management of AR has not been fully confirmed. According to the study by Kočková *et al*. [28], to more accurately predict the timing of surgical intervention for asymptomatic patients with severe AR, the threshold values for LVEDV, LVEDVi, LVESV, and LVESVi should be 281 mL, 124 ${\mathrm{mL/m}}^{2}$, 121 mL, and 56 ${\mathrm{mL/m}}^{2}$, respectively. Furthermore, the authors have also demonstrated that combining volume parameters with quantitative regurgitant parameters can increase the prognostic value (90–95% sensitivity with 78–89% specificity). The research of Harris *et al*. [29] has shown that an LVESVi threshold of 65 ${\mathrm{mL/m}}^{2}$, based on CMR, can better predict the development of cardiac symptoms or the necessity of AVS. The latest European guidelines [8] recommend LV end-systolic parameters to be used as important references in the evaluation of surgical indications for AR. The study conducted by Hashimoto *et al*. [37] in 2022 revealed that among patients with chronic, moderate, or severe AR, those with heart-failure symptoms had a higher LVESVi, measured using CMR, than the asymptomatic or mildly symptomatic patients. Additionally, in the asymptomatic patients or in those under pharmaceutical treatment for mild symptoms, CMR-based LVESVi was found to be independently associated with adverse clinical events, including death and heart failure.

The clinical application of LVEF in the context of AR is limited primarily because it assesses changes in ventricular size rather than myocardial contractility. Due to the powerful compensatory ability of the ventricle, AR patients before stage D may not display a significant decline in LVEF. To date, several CMR techniques have been proposed for early detection of myocardial dysfunction, including strain, LGE, and ECV. Strain imaging allows dynamic assessment of LV function, reflecting the contractility of the myocardial wall. CMR-FT is a technique similar to speckle tracking echocardiography (STE) but with different imaging methods, and can be obtained from SSFP cine sequences and has a better image quality and repeatability than STE [38]. The study by Fernández-Golfín *et al*. [23] showed that LV deformation parameters are superior to the severity of AR, LVEF, and LV volume in predicting the prognosis of patients with AR. The same study also suggested that GLS worsens in the early stage of the disease course and is a very sensitive marker of severe AR; however, global circumferential strain (GCS) and global radial strain (GRS) are responsible for maintaining normal LVEF until advanced stages and can better predict the outcome of aortic valve surgery than GLS. LV strain parameters evaluated via CMR-FT can provide prognostic information for AR patients without prolonging the scanning time. Additionally, this technology is easy to implement in daily clinical practice and thus should be popularized and further studied in patients. LV-pressure and LV-volume overload in AR patients induce myocardial fibrosis (MF), characterized by increased fibronectin and glucosamine deposition and collagen tissue changes [39]. MF is a common feature of many heart diseases and has been linked to increased mortality and other adverse outcomes [40, 41, 42]. MF has been demonstrated in AR patients via myocardial biopsies obtained during active valve surgery [43, 44]. Contrast-enhanced CMR is well established for directly imaging myocardial replacement fibrosis by using LGE. Among the studies we analyzed, the study by Malahfji *et al*. [26] reported that myocardial scar was present in one-third of 392 AR patients they evaluated and was associated with mortality in their multivariable analysis. In another study [21], of the 166 AR patients analyzed, 84 (50.6%) had MF, determined via LGE. In addition, multivariate analysis showed that MF is independently associated with poor medium-term survival and can be used as a prognostic predictor in AR. The latest CMR-T1 mapping technique has proven useful in quantifying extracellular matrix expansion, and interstitial fibrosis assessed using this technique has a good histological correlation with valvular heart disease [45]. The following three T1-mapping-derived metrics have been proposed as markers of increased MF: native T1 time, post-contrast T1 time and myocardial ECV. Myocardial ECV fraction is considered a reliable indicator of MF and is related to the early stage of the disease [46]. To date, the study by Senapati *et al*. [22] is the largest study to evaluate the ventricular cavity and myocardial tissue remodeling in patients with isolated chronic AR by using CMR. Their study found that the incidence of replacement fibrosis in AR is low and not correlated with AR severity. Furthemore, compared with replacement MF and ECV, ECV index (ECVi), which was calculated from ECV multiply by LVEDVi has a stronger correlation with AR severity and adverse clinical outcomes. This is because ECVi represents the absolute total load of LV fibrosis and can better characterize the remodeling changes in cardiomyocyte and extracellular matrix expansion in progressive AR. However, ECV only provides the ratio of extracellular space to total myocardium, concealing the increase in extracellular space under the condition of balanced cellular hypertrophy. Several studies have shown that cellular hypertrophy with interstitial fibrosis occurs before the symptoms in chronic AR, and LV remodeling starts as early as 14 days after the onset of AR, accompanied by MF and extracellular matrix expansion [47, 48, 49]. Therefore, imaging markers derived from LGE and T1-mapping can detect subclinical diseases and myocardial dysfunction before symptoms appear, and can play an important role in the management and risk stratification of chronic AR patients.

Future research on the prognosis of AR should pay extra attention to two aspects. The first is the gender difference in the prognosis of AR patients. Although the research on this issue is limited, related studies have increasingly gained attention. As early as 2002, the existence of this problem was confirmed via animal experiments [50]. Kammerlander *et al*. [51] have demonstrated a clear linear relationship between ARF severity and LV size in men and a relatively less pronounced relationship in women. Moreover, LV remodeling was not obvious in women, implying that women are subjected to surgical intervention later than men and have a relatively worse prognosis. The latest study [52] combining CMR and echocardiography confirmed once again the significant gender difference in LV remodeling in AR patients, revealed that LV-indexed volume is always smaller and LV-indexed inner diameter is significantly larger in women than in men, and pointed out that the error range of echocardiography measurement is more significant in women with larger LV diameter index. Standardized parameters of body surface area may help address the issue, but establishing female-specific standards for ARF and ARV warrants careful consideration. Further research will be needed to determine the pathophysiological mechanisms of passivated LV remodeling in women with chronic AR and to optimize the therapeutic management of female AR patients. Another issue in studies evaluating the prognosis of AR is the BAV. BAV is a congenital heart malformation, with an incidence of 1%–2% in the general population and a male/female ratio of 3:1 [53]. BAV is a common cause of AR and affects LV function and aortic hemodynamics early during the disease course [54]. BAV leads to uneven opening of the aortic valve and eccentric regurgitant jets. In the evaluation of such patients, CMR offers unique advantages, including precise quantification of ARV and ARF, visualization of changes in myocardial morphology and function, as well as assessment of aortic dilation [55]. It has been shown that the BAV group has significantly increased LV volume, aortic diameter, and AR severity than the tricuspid-aortic-valve group. In addition, BAV has been identified to be an independent risk factor for MF, and BAV patients with LGE have a worse prognosis than those without [21]. Therefore, CMR is more valuable than echocardiography for diagnostic, prognostic, and therapeutic assessment in AR patients with BAV.

Although CMR provides additional significant values to AR evaluation, its widespread application still faces several challenges. Firstly, the cost of CMR is relatively high, which increases the economic burden of patients. Furthermore, it is challenging to minimize scan time while obtaining a high spatiotemporal resolution in large volumes of interest. Cardiac arrhythmias pose a challenge, and caution is advised when quantifying the flow in these patients. The CMR technology should be improved for highly accurate imaging and measurement under an irregular rhythm [55]. Additionally, post-processing is time-consuming and affected by user experience, thus delaying the implementation of these techniques into general clinical practice [56]. Finally, although CMR can also be used in assessing cardiac structure and function and monitoring postoperative cardiac remodeling and myocardial changes in postoperative AR patients, related research data are limited and thus cannot provide evidence for the prognostic value of CMR in postoperative AR patients. With the advances in scanning technologies and post-processing software, advanced sequences and methods as well as efficient data collection and analysis strategies will emerge. Meanwhile, the reduction in cost of CMR and its wider application will provide more favorable conditions for clinical practice and research.

## 5. Limitations

The present study has several limitations. Firstly, most of the included studies were single-center observational studies, with limited sample sizes and from tertiary referral centers, and therefore it is difficult to avoid the bias of study subjects. Secondly, the CMR methods, thresholds, and endpoints in the included studies were different, and there were relatively few clinical events. Consequently, statistical limitations hinder further meta-analysis. In addition, advanced technical parameters such as LGE, ECV, and strain were studied individually, causing a lack of robust evidence for the prognostic value of CMR. Finally, there have been very few studies on the use of CMR to follow up postoperative AR patients. Despite the limitations mentioned above, it is important to note that our study still provides valuable insights into the prognostic value of CMR in AR.

## 6. Conclusions

CMR can inform clinicians about multiple parameters, including cardiac size, regurgitant severity, myocardial morphology, and function in the context of AR. CMR-based ARF, LVEDV, and LVESV have significant values in predicting the prognosis of AR patients with AVS as an endpoint. It is recommended to consider using multi-parameter CMR in the clinical management of AR patients for timely interventions and prognostic evaluation. Additional high-quality studies in the future can be used to confirm the prognostic value of CMR-related parameters for AR and to lay a foundation for defining the thresholds of these parameters.

## Abbreviations

AR, aortic regurgitation; CMR, cardiac magnetic resonance; ARF, aortic regurgitation fraction; ARV, aortic regurgitation volume; LVEF, left ventricular ejection fraction; LVEDV, left ventricular end-diastolic volume; LVESV, left ventricular end-systolic volume; HR, hazard ratio; LGE, late gadolinium enhancement; ECV, extracellular volume; GLS, global longitudinal strain; GRS, global radial strain; GCS, global circumferential strain.

## Supplementary Material

Supplementary material associated with this article can be found, in the online version, at https://doi.org/10.31083/j.rcm2412359.
