# Supplementary material for: Prognostic Value of Cardiac Magnetic Resonance Imaging in Chronic Aortic Regurgitation: A Systematic Review and Meta-Analysis
Source: Rev Cardiovasc Med. 2023 Dec 25;24(12):359. doi: 10.31083/j.rcm2412359 (PMC11262437; doi:10.31083/j.rcm2412359)
Supplement: Supplementary file 1 [file 2153-8174-24-12-359-s1.zip › Supplementary Material.docx]

Supplementary Table 1. QUIPS Evaluation of Bias Risk.

| **Author and Year** | **Study Participation** | **Study Attrition** | **Prognostic Factor Measurement** | **Outcome Measurement** | **Study Confounding** | **Statistical Analysis and Reporting** | **Overall Assessment** |
| --- | --- | --- | --- | --- | --- | --- | --- |
| Vejpongsa, P. (2022) | L | L | M | M | L | L | L |
| Zheng, Y. (2021) | L | L | L | M | L | L | L |
| Senapati, A. (2021) | L | L | L | M | L | L | L |
| Fernández- Golfín, C.(2021) | L | L | L | L | L | L | L |
| Faber, M. (2021) | L | L | L | M | M | L | L |
| Postigo, A. (2020) | L | L | L | L | L | L | L |
| Malahfji, M. (2020) | L | L | L | M | M | L | L |
| Seldrum, S. (2019) | M | L | L | M | M | L | M |
| Kockova, R. (2019) | M | L | L | M | L | L | L |
| Harris, A. W. (2017) | M | L | L | M | M | L | M |
| Myerson, S. G. (2012) | L | L | L | M | L | L | L |

QUIPS, Quality in Prognosis Studies; H, high risk of bias; M, moderate risk of bias; L, low risk of bias.


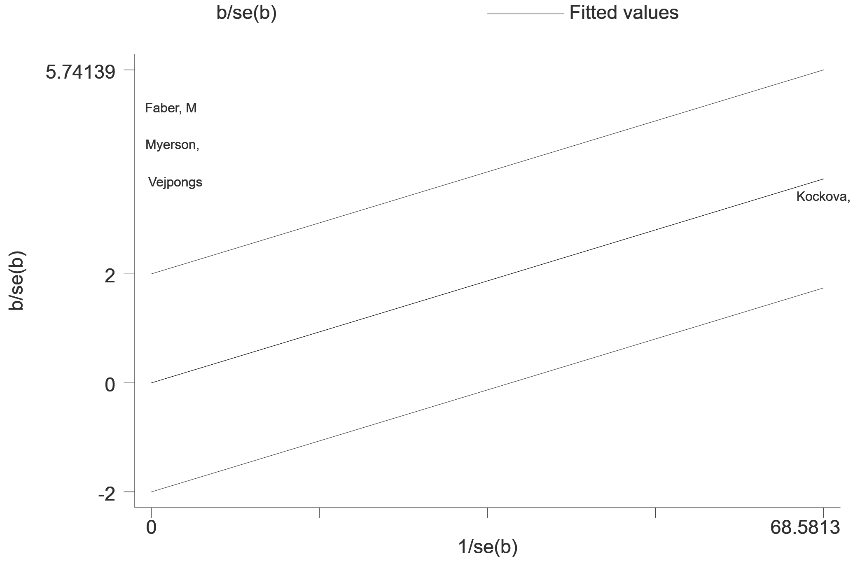

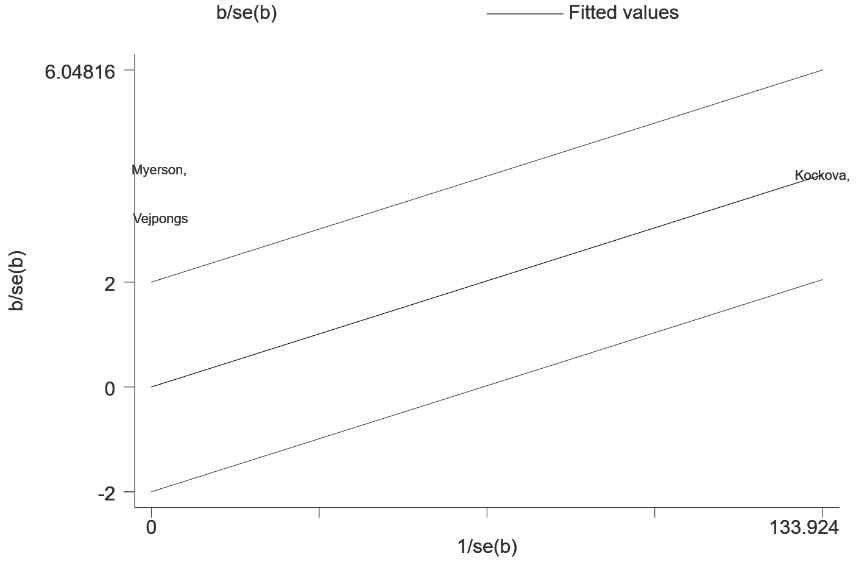


**B**

**A**


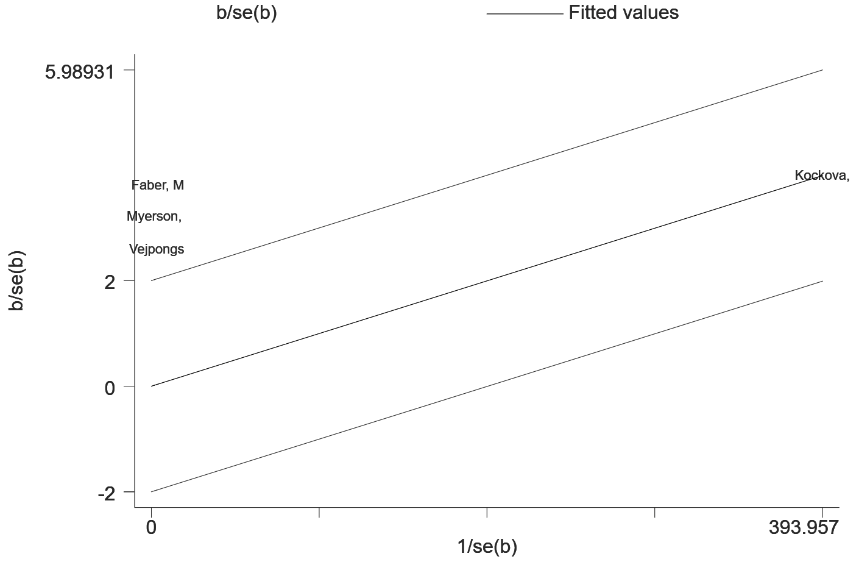

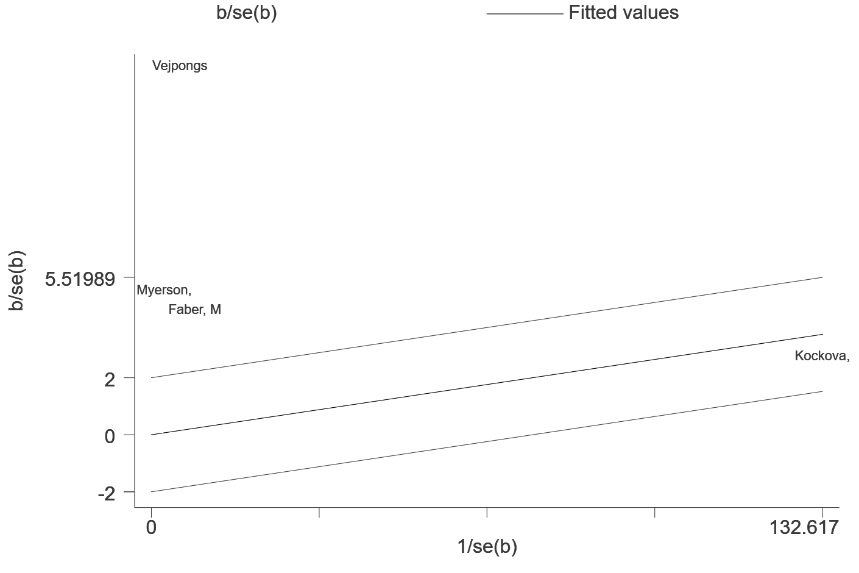


**D**

**C**

**Supplementary Fig. 1. The Origin of Heterogeneity Shown in Galbraith Plot (*I^2^*>50%).** (A) Galbraith plot for aortic regurgitation fraction (ARF); B. Galbraith plot for aortic regurgitation volume (ARV). (B) Galbraith plot for left ventricular end-diastolic volume (LVEDV); D. Galbraith plot for left ventricular end-systolic volume (LVESV).


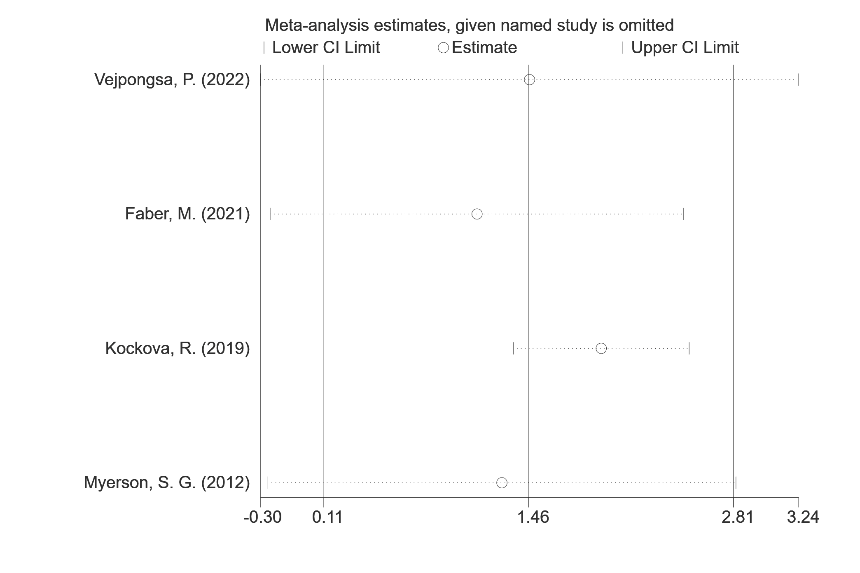

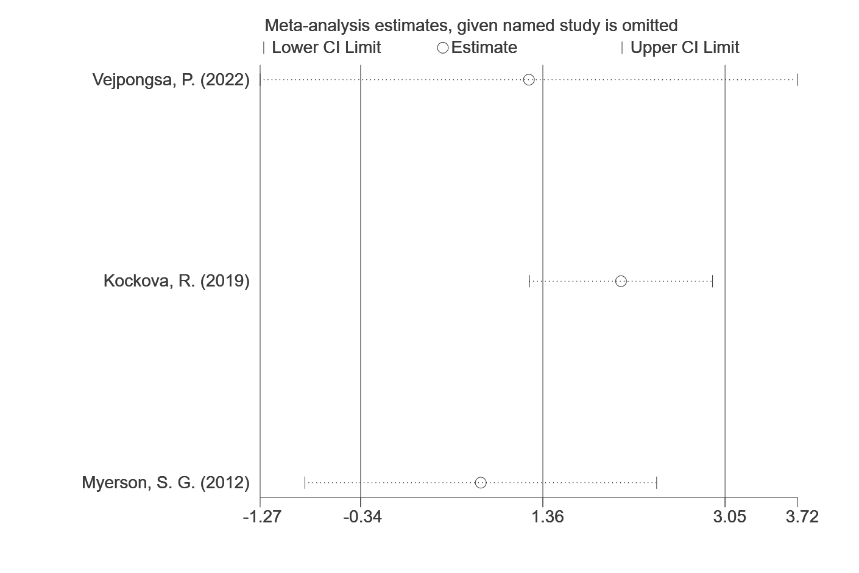


**A**

**B**


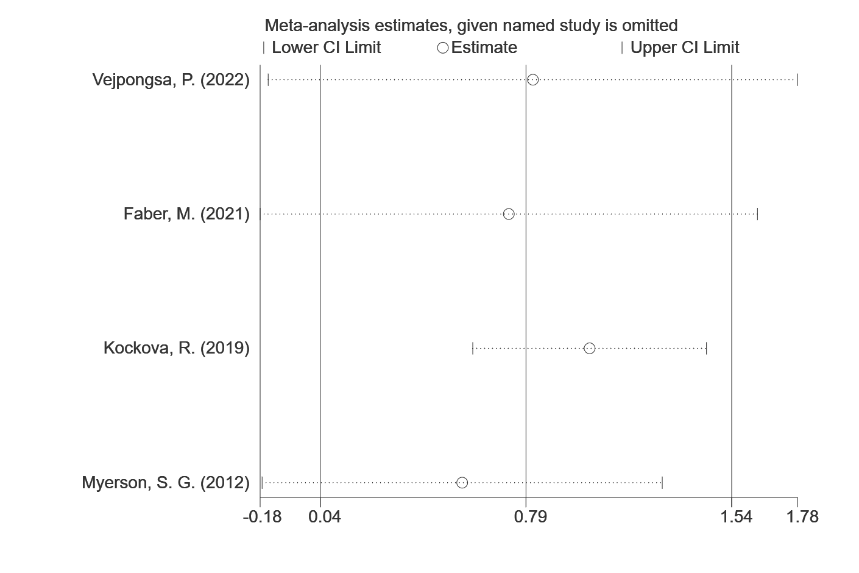

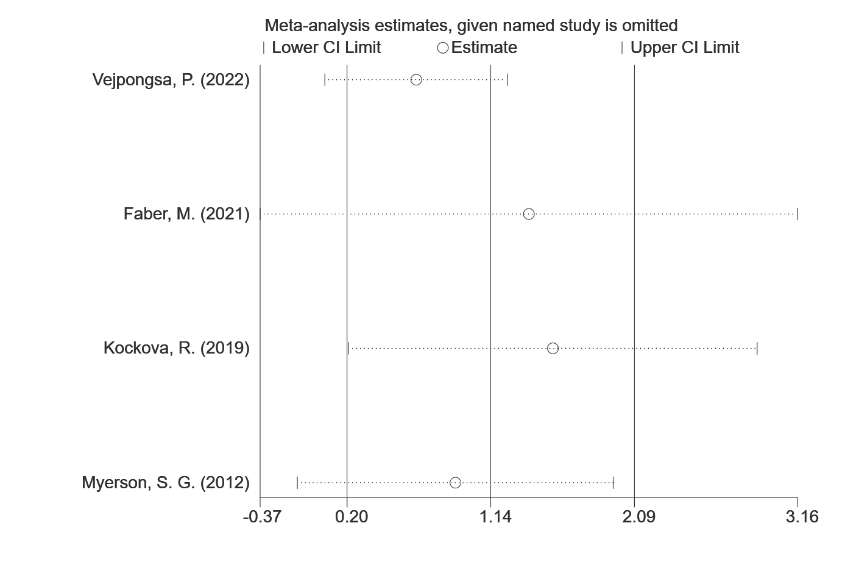


**D**

**C**

**Supplementary Fig. 2. Sensitivity Analysis of CMR Parameters in Predicting Aortic Valve Surgery.** (A) Sensitivity analysis for aortic regurgitation fraction (ARF). (B) Sensitivity analysis for aortic regurgitation volume (ARV). (C) Sensitivity analysis for left ventricular end-diastolic volume (LVEDV); D. Sensitivity analysis for left ventricular end-systolic volume (LVESV).


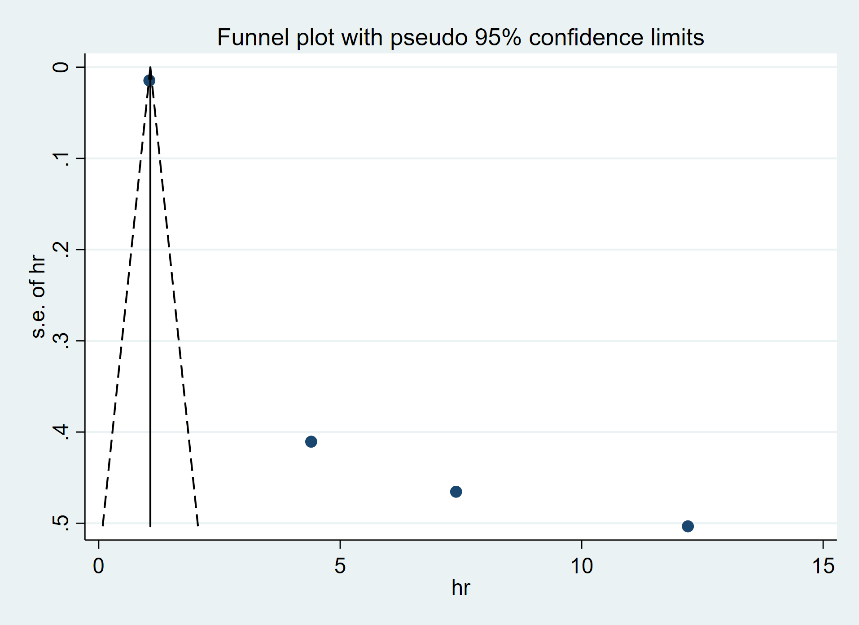

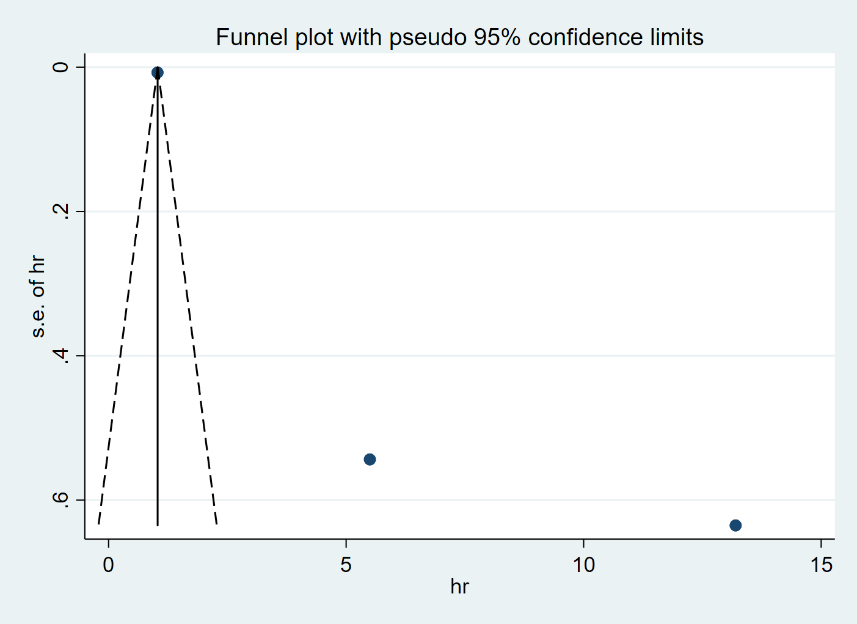


**B**

**A**


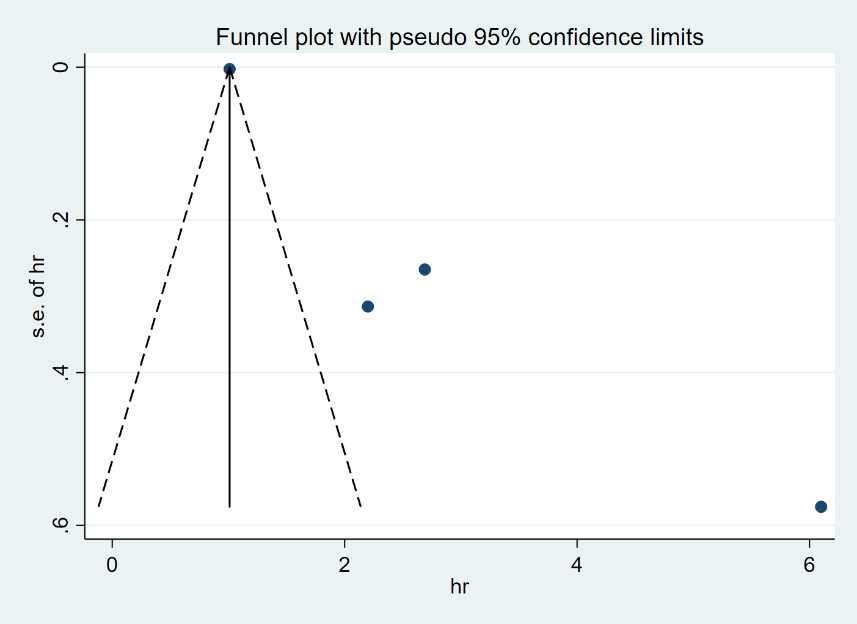

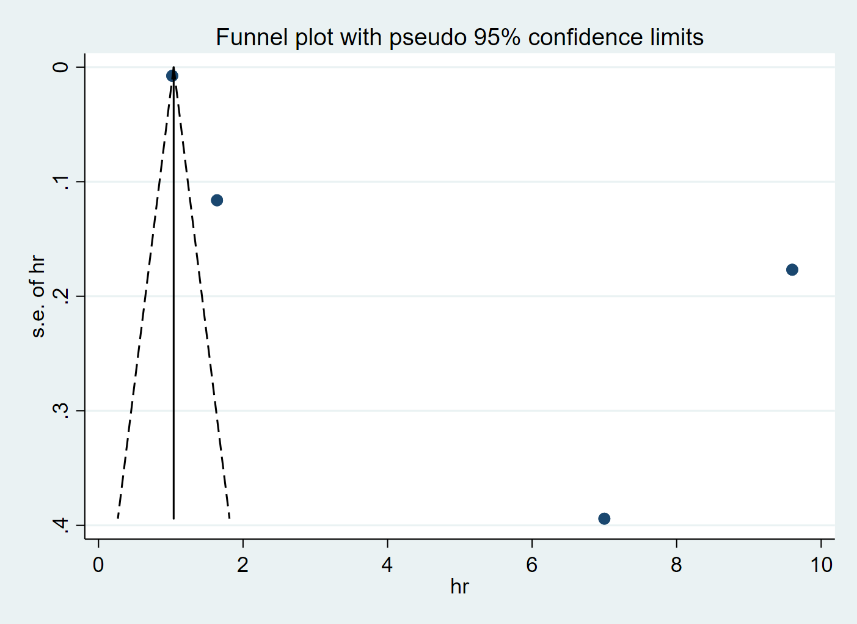


**D**

**C**

**Supplementary Fig. 3.** **Funnel plots of publication bias.** (A) Funnel plot for aortic regurgitation fraction (ARF). (B) Funnel plot for aortic regurgitation volume (ARV). (C) Funnel plot for left ventricular end-diastolic volume (LVEDV); D. Funnel plot for left ventricular end-systolic volume (LVESV).

**Search strategy**

**1.Pubmed**

((((((((((((((magnetic resonance imaging[MeSH Terms]) OR cardiac magnetic resonance imaging[Text Word]) OR MRI[Text Word]) OR CMRI[Text Word]) OR CMR[Text Word]) OR Cardiac MRI[Text Word]) OR Cardiovascular Magnetic Resonance[Text Word]) OR Cardiovascular Magnetic Resonance Imaging[Text Word]) OR CVMR[Text Word]) OR DCMR[Text Word]) OR Cardiovascular MR[Text Word]) OR magnetic resonance[Text Word]) OR MR[Text Word]) AND (((((((((Aortic Valve Insufficiency[MeSH Terms]) OR Aortic Valve Insufficiency[Text Word]) OR Aortic Regurgitation[Text Word]) OR Aortic Valve Regurgitation[Text Word]) OR aortic valve prolapse[Text Word]) OR Aortic Valve Incompetence[Text Word]) OR Aortic Incompetence[Text Word]) OR AR[Text Word]) OR AI[Text Word]))

**2. Cochrane**

#1 MeSH descriptor: [Aortic Valve Insufficiency] explode all trees 104

#2 (Aortic Valve Insufficiency):ti,ab,kw (Word variations have been searched) 252

#3 (Aortic Regurgitation):ti,ab,kw (Word variations have been searched) 528

#4 (Aortic Valve Regurgitation):ti,ab,kw (Word variations have been searched) 477

#5 (Aortic Valve Prolapses):ti,ab,kw (Word variations have been searched) 25

#6 (Aortic Valve Incompetence):ti,ab,kw (Word variations have been searched) 12

#7 (Aortic Incompetence):ti,ab,kw (Word variations have been searched) 16

#8 (AR):ti,ab,kw (Word variations have been searched) 4318

#9 (AI):ti,ab,kw (Word variations have been searched) 5461

#10 #1 OR #2 OR #3 OR #4 OR #5 OR #6 OR #7 OR #8 OR #9 10355

#11 MeSH descriptor: [Magnetic Resonance Imaging] explode all trees 7924

#12 (MRI):ti,ab,kw (Word variations have been searched) 24791

#13 (MR):ti,ab,kw (Word variations have been searched) 6497

#14 (CMR):ti,ab,kw (Word variations have been searched) 979

#15 (cardiac magnetic resonance):ti,ab,kw (Word variations have been searched) 2312

#16 (cardiac magnetic resonance imaging):ti,ab,kw (Word variations have been searched) 1921

#17 (CMRI):ti,ab,kw (Word variations have been searched) 124

#18 (CMRI):ti,ab,kw (Word variations have been searched) 124

#19 (Cardiovascular Magnetic Resonance):ti,ab,kw (Word variations have been searched) 2321

#20 (Cardiovascular Magnetic Resonance Imaging):ti,ab,kw (Word variations have been searched) 1739

#21 (CVMR):ti,ab,kw (Word variations have been searched) 6

#22 (MR):ti,ab,kw (Word variations have been searched) 6497

#23 (DCMR):ti,ab,kw (Word variations have been searched) 1

#24 (Cardiovascular MR):ti,ab,kw (Word variations have been searched) 459

#25 (Cardiovascular MRmagnetic resonance imaging):ti,ab,kw (Word variations have been searched) 0

#26 #11 OR #12 OR #13 OR #14 OR #15 OR #16 OR #17 OR #18 OR #19 OR #20 OR #21 OR #22 OR #23 OR #24 OR# 25 34786

#27 #10 AND #26 430

**3. EMBASE**

('aortic regurgitation'/exp OR 'aortic regurgitation' OR 'aortic regurgitation':ab,ti OR 'aortic valve insufficiency':ab,ti OR 'aortic valve regurgitation':ab,ti OR 'aortic valve prolapses':ab,ti OR 'aortic valve incompetence':ab,ti OR 'aortic incompetence':ab,ti OR ar:ab,ti OR ai:ab,ti) AND ('cardiovascular magnetic resonance'/exp OR 'cardiovascular magnetic resonance':ab,ti OR 'magnetic resonance imaging':ab,ti OR mri:ab,ti OR mr:ab,ti OR cmr:ab,ti OR cmri:ab,ti OR 'cardiac magnetic resonance imaging':ab,ti OR 'cardiac mri':ab,ti OR 'cardiovascular magnetic resonance imaging':ab,ti OR 'cardiovascular mr':ab,ti OR 'cardiac magnetic resonance':ab,ti)

**4. Web of Science**

#1 TS=("Aortic Valve Insufficiency" OR "Aortic Regurgitation" OR "Aortic Valve

Regurgitation" OR "Aortic Valve Prolapses" OR "Aortic Valve Incompetence"

OR "Aortic Incompetence" OR AR OR AI)

Index=SCI-EXPANDED, SSCI, A&HCI, CPCI-S, CPCI-SSH, ESCI, CCR-EXPANDED, IC Time span = all years

#2 TS=("magnetic resonance imaging" OR MRI OR "cardiac magnetic resonance"

OR CMR OR "cardiac magnetic resonance imaging" OR CMRI OR "Cardiac MRI"

OR "Cardiovascular Magnetic Resonance" OR "Cardiovascular Magnetic Resonance

Imaging" OR "Cardiovascular MR")

Index=SCI-EXPANDED, SSCI, A&HCI, CPCI-S, CPCI-SSH, ESCI, CCR-EXPANDED, IC Time span = all years

#3 #1 AND #2
